# The Connexin Mimetic Peptide Gap27 and Cx43-Knockdown Reveal Differential Roles for Connexin43 in Wound Closure Events in Skin Model Systems

**DOI:** 10.3390/ijms19020604

**Published:** 2018-02-18

**Authors:** Chrysovalantou Faniku, Erin O’Shaughnessy, Claire Lorraine, Scott R. Johnstone, Annette Graham, Sebastian Greenhough, Patricia E. M. Martin

**Affiliations:** 1 Department of Life Sciences, School of Health and Life Sciences, Glasgow Caledonian University, Glasgow G4 0BA, UK; Chrysovalantou.Faniku@gcu.ac.uk (C.F.); Erin.OShaughnessy@gcu.ac.uk (E.O.); clairelorraine@hotmail.co.uk (C.L.); srj6n@eservices.virginia.edu (S.R.J.); ann.graham@gcu.ac.uk (A.G.); S.Greenhough@beatson.gla.ac.uk (S.G.); 2 Robert M. Berne Cardiovascular Research Center, University of Virginia School of Medicine, P.O. Box 801394, Charlottesville, VA 22908, USA; 3 Institute of Cardiovascular and Medical Sciences, College of Medical, Veterinary and Life Sciences, University of Glasgow, Glasgow G12 8TT, UK

**Keywords:** wound healing, connexin mimetic peptide, connexin hemichannel, cell migration, SiRNA

## Abstract

In the epidermis, remodelling of Connexin43 is a key event in wound closure. However, controversy between the role of connexin channel and non-channel functions exist. We compared the impact of SiRNA targeted to Connexin43 and the connexin mimetic peptide Gap27 on scrape wound closure rates and hemichannel signalling in adult keratinocytes (AK) and fibroblasts sourced from juvenile foreskin (JFF), human neonatal fibroblasts (HNDF) and adult dermal tissue (ADF). The impact of these agents, following 24 h exposure, on *GJA1* (encoding Connexin43), *Ki67* and *TGF-β1* gene expression, and Connexin43 and pSmad3 protein expression levels, were examined by qPCR and Western Blot respectively. In all cell types Gap27 (100 nM–100 μM) attenuated hemichannel activity. In AK and JFF cells, Gap27 (100 nM–100 μM) enhanced scrape wound closure rates by ~50% but did not influence movement in HNDF or ADF cells. In both JF and AK cells, exposure to Gap27 for 24 h reduced the level of Cx43 protein expression but did not affect the level in ADF and HNDF cells. Connexin43-SiRNA enhanced scrape wound closure in all the cell types under investigation. In HDNF and ADF, Connexin43-SiRNA enhanced cell proliferation rates, with enhanced proliferation also observed following exposure of HDNF to Gap27. By contrast, in JFF and AK cells no changes in proliferation occurred. In JFF cells, Connexin43-SiRNA enhanced *TGF-β1* levels and in JFF and ADF cells both Connexin43-SiRNA and Gap27 enhanced pSmad3 protein expression levels. We conclude that Connexin43 signalling plays an important role in cell migration in keratinocytes and foreskin derived fibroblasts, however, different pathways are evoked and in dermal derived adult and neonatal fibroblasts, inhibition of Connexin43 signalling plays a more significant role in regulating cell proliferation than cell migration.

## 1. Introduction

Connexin43 (Cx43) is expressed in nearly every tissue in the body where it forms hemichannels and intercellular gap junctions and plays diverse roles in coordinating cellular activities [1]. The connexin mimetic peptide (CMP) Gap27, targeted to the SRPTEKTIFFI sequence (amino acids 204–214) on the second extracellular loop of Cx43 is a versatile inhibitor of connexin-mediated communication (CMC) in tissue networks [2,3,4]. Early investigations with these peptides by Evans, Griffiths and colleagues [5,6,7,8] led to advancement of understanding of the role of connexins in the vasculature and identification of heterocellular communication at the myoendothelial gap junction [9,10]. Other studies employing Gap27 in excitable tissue networks have identified the role of connexins in the coordination of cardiomyocyte activities and calcium wave propagation [11,12], at neuronal synapses and more recently in pathological processes such as epilepsy [13]. In non-excitable tissues, Gap27 blocks the passive exchange of small gap junction permeable dyes such as calcein AM and determined a role for intercellular communication during transendothelial migration [14,15].

Many of the ”acute” studies using CMPs have provided evidence for connexin channel signalling in coordinating cellular activities [2]. However, connexins also have reported ”non-channel” functions and controversy exists in longer term studies as to whether channel or non-channel activities play key roles in events such as cell adhesion and migration [16]. This is no less evident in the skin where dynamic changes in connexin expression occur during wound healing [17,18,19]. Antisense oligonucleotides targeted to Cx43 provided the first evidence that connexin based therapies could improve wound healing and resolve inflammation [18,20,21]. Gap27 and other connexin mimetic peptides targeted to the carboxyl terminal domain of Cx43 (such as αCT-1) also improved cell migration rates in 2D and 3D organotypic skin wound model systems [22,23,24,25]. In previous studies [22,23], we determined that while Gap27 enhanced migration rates in keratinocytes and fibroblasts isolated from juvenile foreskin discards it was less effective in fibroblasts isolated from adult dermal explants.

In the present work we compared the effect of Gap27 and SiRNA targeted to Cx43, on cell migration in adult keratinocytes (AK) and adult dermal (ADF), juvenile foreskin (JFF) and neonatal foreskin (HDNF) derived fibroblasts. Our findings provide new insights into the effects of Cx43 channel inhibition versus Cx43 gene expression on cell migration, and our results also show that the response of such behaviour varies between cell types (keratinocyte versus fibroblast) and between cells of the same type (skin fibroblasts) but of different tissue origins.

## 2. Results

### 2.1. The Impact of Gap27 on Cell Migration Rates in Juvenile Foreskin Fibroblasts

**Figure 1:**
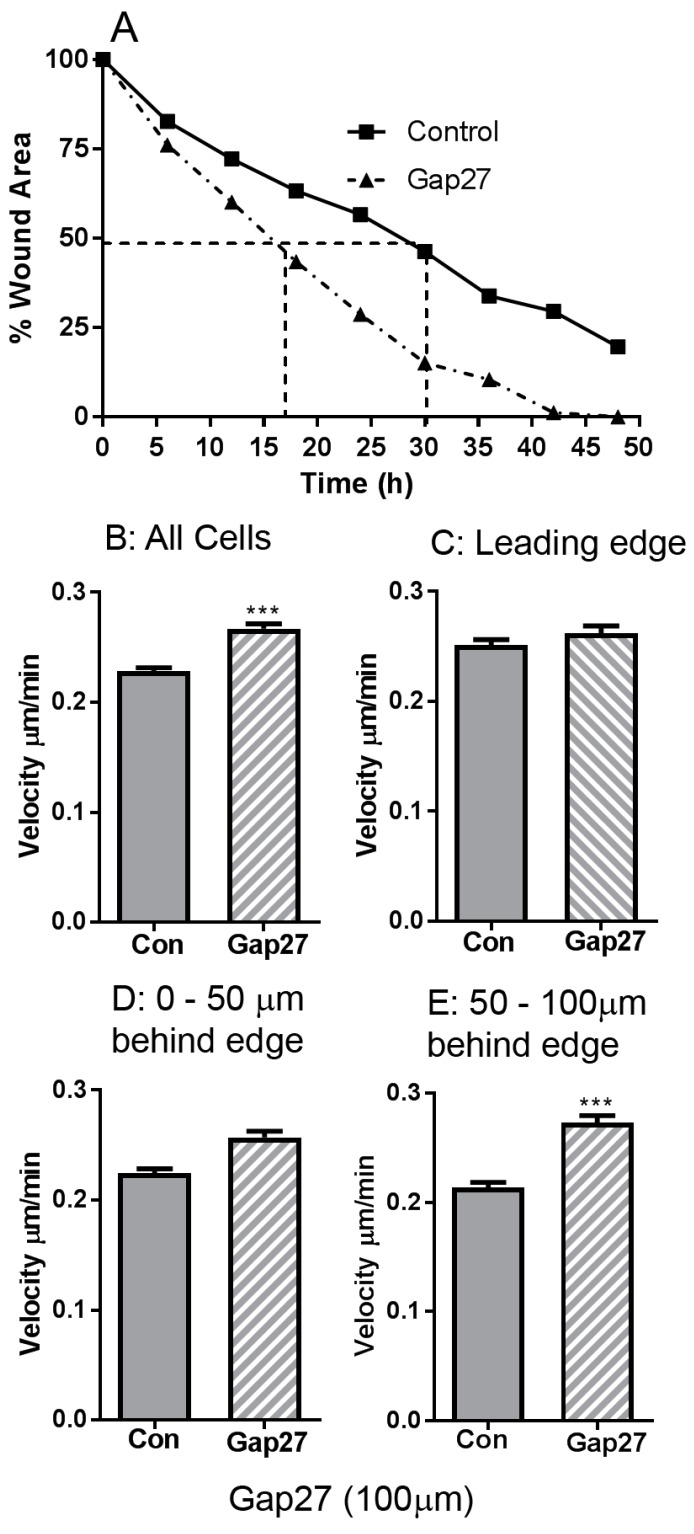
Gap27 (100 μM) influences the speed of Juvenile Foreskin Fibroblasts (JFF) cell migration. (**A**) time-lapse migration data of JFF cells; (**B**) Average cell velocity of JFF cells; (**C**) Cell velocity of JFF cells at the leading edge of the scrape wound; (**D**) Cell velocity of JFF cells 0–50 μm behind the wound edge; (**E**) Cell velocity of JFF cells 50–100 μm behind the wound edge. *n* = 18 cells were tracked in total with 6 cells from each specific area. *** p < 0.005.

Previously we determined that Gap27 inhibits CMC and enhanced scrape wound closure in fibroblasts and keratinocytes derived from juvenile foreskin explants [22,24]. To further explore the effect of 100 μM Gap27 on cell motility, JFF cells were subject to time-lapse microscopy and images captured every 15 min over a 48 h migration period. The speed of cell movement in non-treated and Gap27 (100 μM) treated cells was analysed: Gap27 treated cells reached 50% scrape closure in approximately half the time taken by non-treated cells (Figure 1A).

Image trajectory analysis was performed to elucidate if the differences observed in JFF scrape closure ± Gap27 were due to variation in cell directionality or speed of movement. Graphical representation of the XY co-ordinate data obtained from tracking the movement of 18 individual cells for each set of JFF images (±100 μM Gap27) illustrated differences in cell migration between the control and peptide treated cells, most noticeably an increase in distance travelled by the Gap27 treated cells, compared to controls, into the scraped area. The data also suggest that the majority of peptide treated cells migrate in straighter lines towards the scraped area compared to controls which had a more lateral movement. Cell tracking data determined that Gap27 treatment in JFF cells significantly increased the average cell velocity over 48 h by 2.5 μm/h compared to controls; the average velocity was 0.23 ± 0.003 μm/min (13.8 μm/h) in control cells and 0.27 ± 0.004 μm/min (16.3 μm/h) in peptide treated cells (Figure 1B). To further explore these differences, data sets of the rate of cell movement at the leading edge (Figure 1C), 0–50 μm (Figure 1D) and 50–100 μm (Figure 1E) behind the wound edge were analysed. At the leading wound edge, the migration of control and peptide treated cells were comparable (Figure 1C). However, in cells located 0–50 μm behind the wound edge cell migration rates in Gap27 was faster, with the greatest difference in velocity occurring 50–100 μm behind the wound edge, where the Gap27 treated cells migrated at a rate of 0.273 ± 0.006 μm/min compared to the non-treated cells that migrated at rates of 0.214 ± 0.004 μm/min (Figure 1D,E). These data indicate that during scrape wound closure in JFF cell monolayers, cell velocity is greater in wound edge cells compared to cells behind the wound edge. However, Gap27 treatment enhances the scrape wound closure in JFF cell monolayers in vitro by increasing cell velocity in cells behind the wound edge.

### 2.2. Impact of Gap27 and SiRNA Targeted to Cx43 on Cell Migration in Skin Model Systems

**Figure 2:**
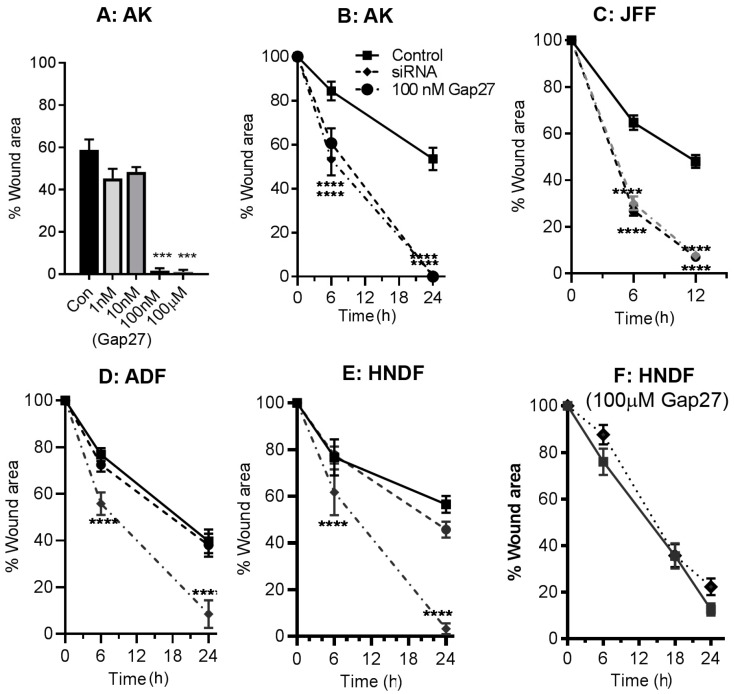
Gap27 and SiRNA targeted to Cx43 have differential effects on scrape wound closure rates in skin cells. Dose response of Gap27 in AK cells (**A**); SiRNA targeted to Cx43 and 100 nM Gap27 enhance scrape wound closure in AK (**B**) and JFF cells (**C**); Gap27 does not enhance cell migration rates in ADF or HNDF cells (**D**–**F**). *n* = 3, ** *p* < 0.01; *** *p* < 0.005.

While both CMPs and antisense Cx43 knockdown strategies are widely accepted to enhance wound closure rates [20,22,26], a direct comparison of their effects on cell migration events has not been reported. We thus explored the impact of Gap27 and SiRNA targeted to Cx43 on scrape wound closure rates in keratinocytes and fibroblasts isolated from adult skin biopsies and compared this to cells derived from juvenile foreskin and neonatal human fibroblasts. Initially a dose response of Gap27 determined that the peptide effectively enhanced scrape wound closure rates in primary adult keratinocytes at 100 nM–100 μM, but was without effect at lower doses (Figure 2A). In these AK cells, SiRNA targeted to Cx43 significantly enhanced the rate of scrape wound closure (Figure 2B). In JFF cells, 100 nM Gap27 and SiRNA targeted to Cx43 significantly enhanced the rate of scrape wound closure with 50% closure rates more than two times faster than non-treated samples (Figure 2C). Multiple studies performed in adult fibroblasts demonstrated that 100 nM–100 μM Gap27 had limited impact on cell migration responses (Figure 2D); by contrast, significant increase in 50% closure rates occurred in adult fibroblasts transfected with SiRNA targeted to Cx43, compared with the SiRNA control (Figure 2D).

To further compare the efficacy of Gap27 on fibroblast cell migration events, scrape wound assays were also performed using commercially sourced human neonatal dermal fibroblasts. Treatment with Gap27 was ineffective in enhancing migration responses at concentrations of 100 nM or 100 μM (Figure 2E,F). By contrast, inhibition of Cx43 expression by transfecting the cells with SiRNA targeted to Cx43 significantly improved cell migration rates suggesting a ”non-channel” role for Cx43 in migration of these neonatal fibroblasts (Figure 2E). We also explored the migration responses of HNDF cells on various extracellular matrix components in the presence and absence of 100 μM Gap27. Although the cells migrated slightly faster on both fibronectin and collagen matrices, Gap27 still did not enhance cell migration rates [27].

### 2.3. Gap27 Attenuates Hemichannel Signalling at Lower Doses than Gap Junction Coupling

**Figure 3:**
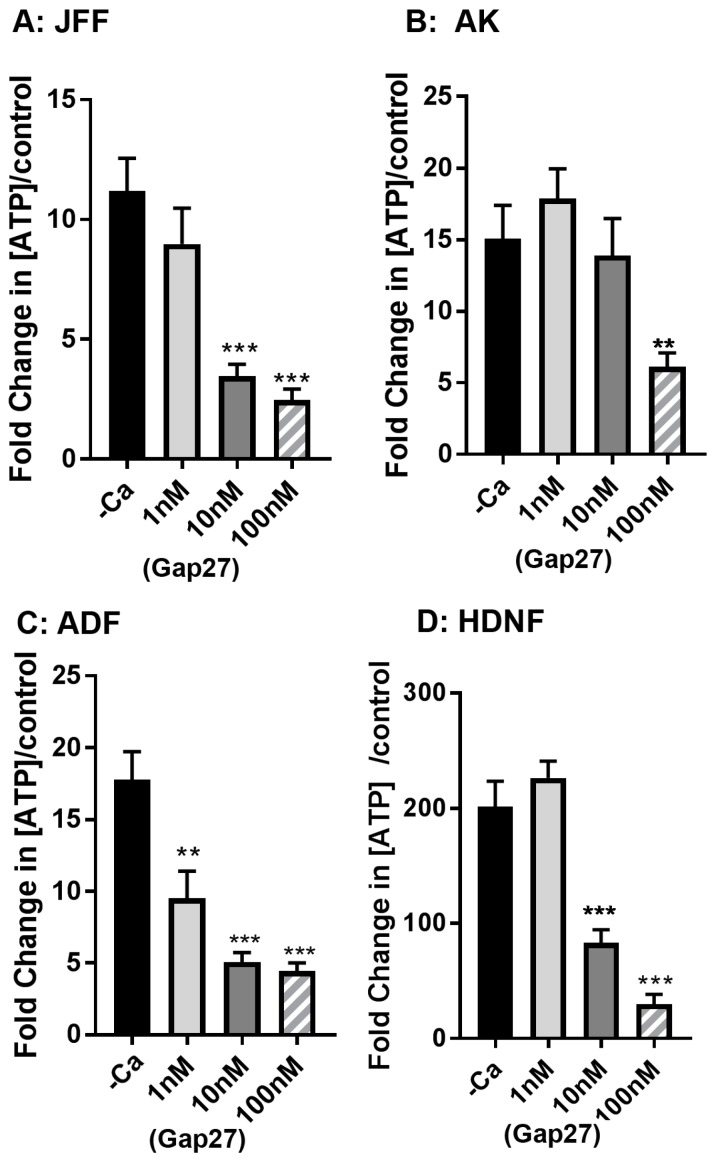
Gap27 inhibits hemichannel signalling. JFF cells (**A**); Keratinocytes (**B**); ADF (**C**) and HNDF (**D**) cells were exposed to a dose response of Gap27 and ATP release assays performed following calcium deprivation. Data are presented at the Fold change in ATP release over control cells that were not subject to calcium challenge. *n* = 3, *** *p* < 0.005, ** *p* < 0.01.

In previous studies, microinjection analysis with Alexa 488 determined that Gap27 effectively inhibits gap junction coupling at concentrations of 50 μM in keratinocytes and HeLa43 cells [23,24]. We also previously reported that Gap27 inhibits ATP release in both keratinocytes and fibroblasts isolated from juvenile foreskin tissue discards at concentrations of 100 μM [22,23,28]. In view of the stark contrast in the impact of Gap27 on cell migration rates between the different cell types, we further explored the ability of Gap27 to attenuate hemichannel activity. In all the cell types, Gap27 effectively inhibited ATP release in a dose responsive manner, effective at 10–100 μM concentrations (Figure 3A–D). This data further suggests that in the ADF and HNDF, hemichannel signalling events are unlikely to be involved in controlling cell migration.

### 2.4. The Impact of Gap27 and SiRNA Targeted to Cx43 on Gene and Protein Expression Profiles in Skin Model Systems

At the end point of the cell migration assays, RNA and protein were extracted and subject to qPCR and Western blot analysis to determine any significant changes in gene and or protein expression profiles.

**Figure 4:**
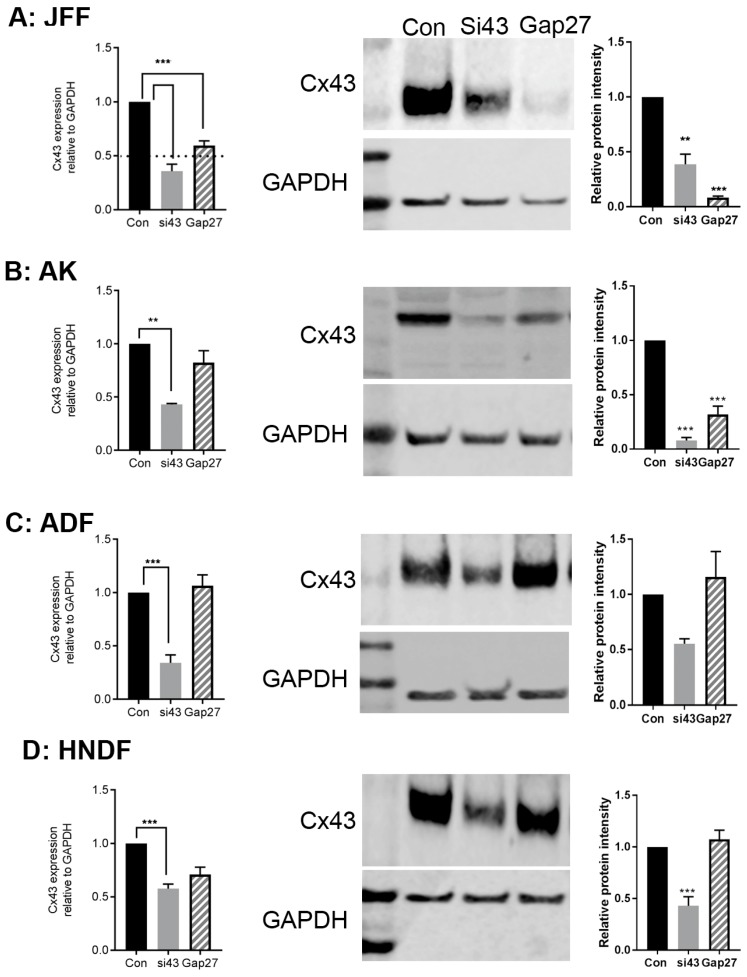
The impact of Gap27 and SiRNA targeted to Cx43 on gene expression. At the end of scrape wound closure assays, RNA and protein was harvested from cells and subject to real time PCR or Western blot analysis to determine levels of Cx43 expression. JFF (**A**); AK (**B**); AF (**C**) and HNDF (**D**). Panel 1 represents changes in gene expression, Panel 2 represents a typical Western blot, Panel 3 represents densitometric analysis of three Western blots. *n* = 3, *** *p* < 0.005, ** *p* < 0.01 a threshold of two fold increase or decrease in expression was considered significant.

SiRNA targeted to Cx43 reduced the level of *Cx43* gene and protein expression by >50% in JFF, AK, ADF and HNDF cells (Figure 4A–D). Exposure to 100 nM Gap27 for up to 24 h reduced *Cx43* gene expression levels in JFF cells but had limited impact on *Cx43* gene expression levels in the other cell types (Figure 4A–D (panel 1)). In ADF and HNDF cells, 100 nM Gap27 did not influence the level of Cx43 protein expression (Figure 4C,D (panels 2 and 3)). However, in JFF cells exposure to 100 nM Gap27 for 24 h caused a >2-fold reduction in the level of Cx43 protein expression and a similar trend, although not as pronounced, was observed in AK cells (Figure 4A,B (panels 2 and 3)).

### 2.5. The Impact of Gap27 and SiRNA Targeted to Cx43 on Cell Proliferation, TGF-β1 and SMAD3 Signalling Pathways

**Figure 5:**
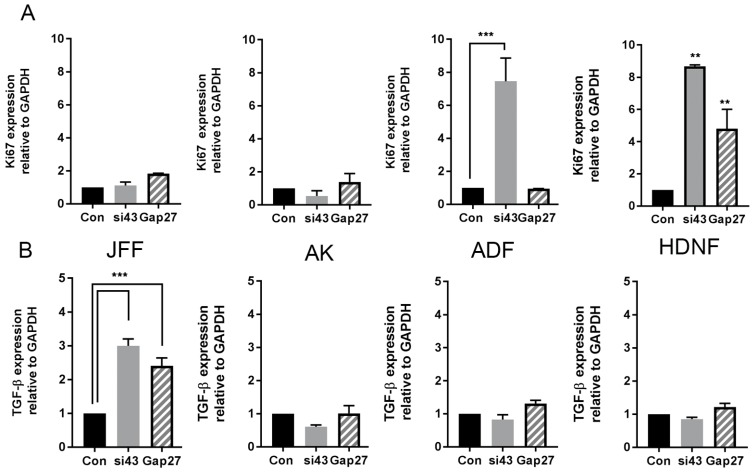
The impact of Gap27 and SiRNA targeted to Cx43 on *Ki67* and *TGF-β1* gene expression. At the end of scrape wound closure assays RNA was harvested from cells and subject to real time PCR analysis to determine changes in gene expression of (**A**) *Ki67* and (**B**) *TGF-β1 n* = 3, *** *p* < 0.005, ** *p* < 0.01 a threshold of two fold increase or decrease in gene expression was considered significant.

To determine if the differences observed in cell migration responses between the various cell groups were related to changes in cell proliferation, the level of *Ki67* gene expression in each of the cell types and treatment groups was determined. In JFF and AK cells, none of the treatments evoked a greater than two-fold increase in the level of *Ki67* gene expression (Figure 5A, AK and JFF panels). By contrast, in ADF cells a 10-fold increase in *Ki67* gene expression was observed in cells exposed to SiRNA targeted to Cx43 but not in those exposed to Gap27 (Figure 5A, ADF panel). In HNDF cells, proliferation was dramatically enhanced in all treatment groups (5-10 fold) compared to non-treated cells (Figure 5A, HNDF panel).

Transforming growth factor β1 (TGF-β1) is a major transcription factor regulating cell signalling events involved in migration. The level of *TGF-β1* gene expression was enhanced ~3–4 fold in JFF cells following knockdown of *Cx43* gene expression and following treatment with 100 nM Gap27 for 24 h (Figure 5B, JFF panel). By contrast, in AK, AF and HNDF cells, Gap27 and SiRNA targeted to Cx43 had limited impact on the level of *TGF-β1* gene expression levels (Figure 5B) at the 24 h time point.

**Figure 6:**
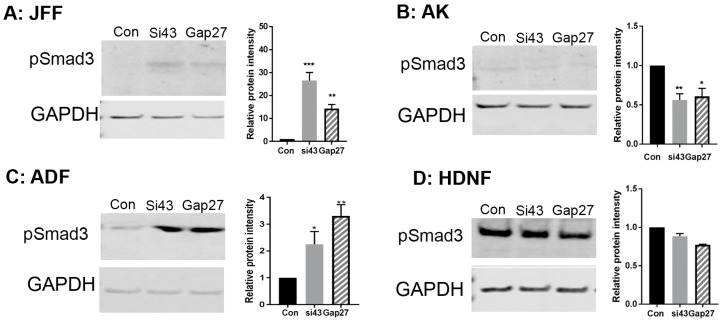
The impact of Gap27 and SiRNA targeted to Cx43 on pSmad3 expression. At the end of scrape, wound closure assays protein was harvested from cells and subject to Western blot analysis. Blots were probed with an antibody targeted to pSmad3 and GAPDH. Representative blots are presented for each cell type JFF (**A**); AK (**B**); AF (**C**) and HNDF (**D**) (panel 1). Panel 2 represents densitometric analysis of triplicate blots and relative pSmad3 protein levels compared to GAPDH. *n* = 3, *** *p* < 0.005, ** *p* < 0.01; * *p* < 0.5.

Finally, previous reports identified that phosphorylation of Smad3 is associated with exposure of mucosal derived fibroblasts to Gap27 [29]. Probing the Western blots with an antibody targeting pSmad3 identified that in JFF and ADF cells exposure to SiRNA targeted to Cx43 and 100 nM Gap27 for 24 h enhanced the level of pSmad3 expression (Figure 6A,C). By contrast, in HNDF and AK cells the level remained constant (Figure 6B,D), further suggesting differential signalling pathways are triggered in different compartments of the skin following remodelling of Cx43.

A summary of the combined data is presented in Table 1.

## 3. Discussion

In the present study we compared the effect of Gap27 and SiRNA targeted to Cx43 on scrape wound closure rates in fibroblasts derived from neonatal, juvenile foreskin and adult dermal explants and matched adult keratinocytes. Both of these reagents enhanced scrape wound closure in the JFF cells and adult keratinocytes with Gap27 enhancing cell migration rates at concentrations of 100 nM, reflecting the dose of peptide that inhibited hemichannel signalling and supporting the concept that ATP release via hemichannels is required for keratinocyte galvanotaxis [30]. By contrast, in the AF and HNDF cells, SiRNA targeted to Cx43 enhanced scrape wound closure, but treatment with Gap27 was without effect, extending studies where we reported limited effects of Gap27 on adult fibroblast migration rates [22,23]. Profound differences in cell migration, proliferation and the TGF-β1/pSmad3 signalling axis occurred between the cells isolated from different skin compartments. The data provides new insights into the controversy surrounding Cx43 channel versus non-channel functions in cell migration and wound repair responses [16] and suggests that inhibition of hemichannel signalling alone is insufficient to modify cell migration events in adult dermal fibroblasts.

Within epithelial tissues, including the skin and cornea, it is widely accepted that downregulation of Cx43 is favourable to wound closure, as reported in the skin of connexin-deficient mice [19] and by the development of Cx43 anti-sense oligonucleotides, that have proven effective in rat models and in human clinical trials [18,26,31,32]. Other studies by Gourdie and colleagues used a peptide targeting the carboxyl tail of Cx43 and its binding site with the PDZ domain to improve wound healing, resolve inflammation and reduce scarring in rat models and recently in human clinical trials [33,34,35]. Studies using Pep5, based on the Gap27 sequence, have shown remarkable effects on tissue repair and inflammation in the retina, cornea and spinal cord [36,37,38]. Recent studies using Gap27 have also shown that this peptide is effective in improving rabbit corneal wound healing [39] and in primary human gingival fibroblasts, isolated from donors aged 26–48 years of age [29]. A further peptide TAT-Gap19, has also recently been reported to enhance scrape wound closure of human gingival fibroblasts [40]. TAT-Gap19, targeted to the intracellular loop of Cx43, was designed as a cell permeant peptide and has been extensively used to characterise interactions between the intracellular loop of Cx43 and the carboxy terminal tail [41,42]. This peptide effectively blocks hemichannel activity but has no effect on gap junction coupling, in contrast to Gap27 which blocks all forms of connexin mediated communication. Thus, inhibiting *Cx43* gene expression and blocking channel function both have a positive influence on wound closure events; however, comparisons of the mechanisms underlying the modes of action of these different means of remodelling Cx43 remains unresolved.

In the present study we have identified profound differences in wound healing events in skin cells isolated from different sources, and between adult epidermal and stromal derived fibroblasts, which relate to whether Cx43 channel function or gene expression is regulated.

In the case of JFF cells, derived from juvenile foreskin discards (a thin layer of tissue) and in adult keratinocytes, both Gap27 and SiRNA targeted to Cx43 enhanced scrape wound closure. Further, in both of these cell types at the end point of the migration time course, neither knockdown of Cx43 expression or inhibition of channel function by Gap27 had any effect on cell proliferation as monitored by *Ki67* gene expression. This re-enforces our previous findings where irradiated juvenile fibroblasts were used and Gap27 effectively enhanced wound closure [22]. Hence in JFF cells and keratinocytes proliferation factors are not involved in the enhanced cell migration response, suggesting hemichannel signalling plays an important role in co-ordinating cellular responses [30,40].

By contrast, in ADF and HNDF while SiRNA targeted to Cx43 accelerated the rates of scrape wound closure, exposure to Gap27 had a limited effect on cell migration rates. In AF cells, Gap27 did not influence the gene expression of *Ki67* but decreasing *Cx43* gene expression enhanced cell proliferation. In the HNDF cells, exposure to Gap27 and to SiRNA targeted to Cx43 enhanced cell proliferation, but only SiRNA targeted to Cx43 enhanced cell migration rates. It is also noteworthy that at the end point of the migration assays, while SiRNA targeted to Cx43 reduced Cx43 gene and protein expression in all cell types by >50% exposure to Gap27 had no effect on Cx43 protein levels in AF cells. By contrast, in the JFF and AK cells Gap27 reduced the level of Cx43 protein expression by ~50%, however it had a limited effect on *Cx43* gene expression, suggesting Cx43 changes were post-transcriptional. A similar effect was also observed in adult human gingival fibroblasts where Gap27 also enhanced wound closure rates [29]. Studies using mouse NIH3T3 fibroblasts also determined that Gap27 reduced Cx43 protein expression in line with our observations in JFF cells [43]. Studies in our lab also determined that Gap27 improved cell migration rates in primary neonatal mouse fibroblasts [27] and keratinocytes [44]. Taken together, this data suggests that in human adult dermal fibroblasts and neonatal fibroblasts, non-channel functions of Cx43 may be more important than acute hemichannel signalling in regulating cell migratory behaviour.

Previously, we identified changes in expression of a panel of genes associated with extracellular matrix (ECM) deposition in JFF cells following exposure to Gap27, including metalloproteinase 9 (MMP-9) and connective tissue growth factor (CTGF) [23]. Several other reports have indicated a link between altered Cx43 expression and ECM regulation, including in fibroblasts isolated from a patient harbouring a non-functional Cx43 mutation associated with the Cx-channelopathy oculodentodigital dysplasia [45]. Modifying Cx43 expression or function by antisense oligonucleotides and peptide αCTI have also been associated with alterations in ECM deposition [20,33]. Further studies by Tarzemany et al. systematically reviewed the impact of Gap27 and more recently TAT-Gap19 on gene expression and cell signalling pathways involved in wound closure events in human adult gingival fibroblasts [29,40]. Both peptides effectively enhanced wound closure rates and gene array analysis after 24 h indicated changes in expression of a panel of genes related to ECM deposition including a number of MMP proteins and CTGF, in agreement with our previous studies on JFF cells [23].

The TGF-β signalling pathway is important in controlling cell migration events, and a number of studies have suggested links between TGF-β1 and Cx43 expression in wound healing scenarios [20,46]. Treatment with either Gap27 or SiRNA targeted to Cx43 enhanced the gene expression of *TGF-β1* in JFF cells, but had little impact following 24 h exposure to these reagents in the adult keratinocytes and fibroblasts. However, it remains possible that expression of *TGF-β1* is transiently induced at earlier time points, since TGF-β1 has been reported to stimulate chemotactic migration of human fibroblasts [47].

The TGF-β/Smad3 signalling axis plays an important role in cell migration events, and has been linked with Cx43 expression. In line with this in both JFF and ADF cells, levels of pSmad3 were increased following 24 h exposure to Gap27 and SiRNA targeted to Cx43. A profound increase in pSmad3 was also reported in gingival fibroblasts exposed to Gap27 for 24 h [29]. Smad3 and the carboxyl terminal tail of Cx43 both compete for a similar ‘microtubule binding domain’ [48]. Thus, alteration in Cx43 expression levels or function may modify the interaction. Upon translocation to the nucleus, Smad3 is phosphorylated to pSmad3 and exerts transcriptional control on a range of genes involved in regulation of inflammation, cell proliferation and re-epithelisation in both positive and negative tissue-specific ways. Given the diverse pathways that pSmad3 can regulate, it is highly likely that in the ADF cells, where Gap27 did not influence cell migration, other key cellular events may be affected. Although no evidence of induction of pSmad3 expression was observed in the keratinocytes, a host of other signalling pathways including the ERK1/2 and JNK pathways may be influenced [49] and this is subject to further investigation. In the present studies, the influence of modulation of Cx43 was assessed in monocultures. In the future it will be important to exploit our 3D organotypic models as it is well established that keratinocytes and fibroblasts can influence responses of adjacent cells as part of a coordinated tissue event [50].

## 4. Materials and Methods

### 4.1. Cell Culture

Primary juvenile human dermal fibroblasts (JFF cells) were derived from paediatric foreskins discarded at surgery following informed consent with ethical approval by Yorkhill Hospital Trust Research Ethics Committee, Glasgow, or were kindly gifted by Prof J Brandner, University Hamburg, with their use approved by the ethics committee of the Aerztekammer Hamburg (060900) as previously described [22]. Human neonatal dermal fibroblasts (HNDF) were sourced from Invitrogen (Cat No.: C0045C, Paisley, UK) and human adult dermal fibroblasts (AF) and keratinocytes (AK) were obtained from the GCU Skin Research Tissue Bank, which has NHS and GCU research ethical approval (NHS REC Ref 16/ES/0069). Fibroblasts and keratinocytes were isolated from tissue explants and cultured as previously described [22,24]. All cells were maintained at 37 °C, 5% CO_2_. Monolayers of all fibroblasts were maintained in DMEM (Lonza, Wokingham, UK) supplemented with 10% (*v*/*v*) foetal calf serum, 2 mM glutamine, 50 Units/mL penicillin/streptomycin (Lonza, Wokingham, UK), hereafter termed ”complete” DMEM (cDMEM). Keratinocytes were maintained in EPILIFE medium as previously described [51] (Thermo Fisher, Paisley, UK). Cells were seeded at appropriate densities on 24-well plates for ATP assays (~0.5 × 10^5^ per well) or 6-well plates (~1 × 10^6^ cells per well) for all other assays.

### 4.2. Inhibition of Connexin Mediated Communication

For the purpose of this study, Gap27 (MW 1305) (Zealand Pharma, Glostrop, Denmark) was used in aqueous solution at doses ranging from 1–100 μM for 15 min–48 h depending on experimental requirements. The peptide and media were replaced at 8 h intervals.

### 4.3. Knockdown of Cx43 Expression by siRNA

SiRNA duplex sequences targeted to Cx43 (TriFECTa^®^RNAi Kit from Integrated DNA Technologies (Tyne & Wear, UK)) along with a fluorescently-labelled scrambled transfection control duplex: TYE 563™ was used to knockdown Cx43 expression. SiRNA transfection was carried out using Lipofectamine 3000 transfection reagent (Invitrogen, Paisley, UK). Transfection reagents and siRNAs were combined and incubated for 20 min at room temperature for complex formation. Cells were transfected with a final concentration of 5 nM siRNA diluted in 1.5 mL of EPILIFE medium for primary keratinocytes and serum free DMEM (SFM) for fibroblasts. Twenty hours post transfection scrape wound assays were performed and cells subsequently harvested for endpoint analysis as described below. Transfection efficiency of control SiRNA, determined by fluorescent microscopy analysis of the scrambled SiRNA control was ~90% for all cell types.

### 4.4. Hemichannel Functionality Assays

Hemichannel activity was assessed by ATP release assays with minor modifications to that previously described [51]. Briefly, cells were seeded on 24-well plates and grown to ~80% confluency overnight. Cells were then washed three times in SFM and incubated in SFM for 1 h prior to exposure to Gap27 for 90 min. Following this, one half of the plate was challenged with Ca^2+^ and Mg^2+^ free PBS in the presence or absence of Gap27 for 15 min. Supernatants were collected and microcentrifuged at 10,000 rpm for 5 min prior to addition of 25 μL of supernatant to a well of an opaque-walled Nunc 96 well plate containing 25 µL ATP assay mix diluted 1:10 with ATP dilution buffer (Sigma-Aldrich, Gillingham, UK). ATP standards (25 µL) 0–10 nM diluted in SFM, and in PBS, were also added in duplicate. Luminescence was measured in relative luminescence units (RLU) using the Fluostar Optima plate reader (BMG Labtech, Aylesbury, UK). Experiments were performed in triplicate per treatment group, and each experiment was carried out a minimum of three times (*n* = 3). Data is represented as the fold change in ATP released between control and peptide treated wells.

### 4.5. Scrape Wound Assays and Time-Lapse Microscopic Analysis of Cell Migration

Cells were pre-exposed to peptide or SFM for 90 min, or were transfected with SiRNA targeting Cx43 for 20 h, prior to introducing a scrape wound to confluent cell monolayers using a sterile 100 µL pipette tip. Cell migration was monitored by taking triplicate images of wound area 0, 6, 12 and 24 h post scraping on a CMEX-3200 camera [51]. The scrape wound area was measured at each time point using Image J software. Values were normalised by comparing with the corresponding initial wound size. For time-lapse microscopy analysis, images were recorded on a Zeiss Axiovert 100 microscope (Cambridge, UK) linked up to a CCD camera (Nikon Eclipse TS10, Kingston Upon Thames, UK). Image capture was controlled by AQMsoftware (Kinetic Imaging Ltd., Nottingham, UK). Images were captured every 15 min for up to 48 h [52]. The movement of 18 individual cells for each set of JFF time-lapse images in the presence or absence of 100 μM Gap27, were tracked using Image J software tracking plug-in. The size of each image was 512 × 512 pixels with a diameter of ~500 μm, therefore, at ×100 magnification each pixel represented approximately 1 μm. This value was used as the x/y calibration value with a time interval value of 15 min. Cells were tracked by clicking on the leading edge of a cell on sequential images representing every 15 min over the 48 h period. Six cells were randomly chosen from each of (1) wound edge; (2) 0–50 μm from the wound edge and (3) 50–100 μm from the wound edge. The data output produced by Image J software included the XY co-ordinates together with distance and velocity values. The XY co-ordinates were plotted on a graph using Excel software, providing an individual track for each cell, enabling visualisation of cell movement over 48 h.

### 4.6. RNA Extraction and Real Time PCR

The Bioline ISOLATE RNA Kit (Bioline, London, UK) was used according to manufacturer’s instructions to extract RNA from cell monolayers (usually 2 wells for a 6 well plate). RNA concentrations were determined using a Nanodrop ND-100 at 260/280 nm. cDNA was prepared from the RNA samples using cDNA synthesis kit from Primerdesign and real-time PCR was performed using Primerdesign Master Mix kit (Primer Design, Chandlers Ford, UK). Primers amplifying human *Cx43*, human *Ki67*, human *TGF-β1* and the house keeping gene *GAPDH* were purchased from IDT (Tyne & Wear, UK) (Table S1). All reactions were performed in an ABI 7500 FA Real-Time PCR system (Applied Biosystems, Warrington, UK). The mRNA expression level for each gene was determined using the Δ*C*_t_ method and each sample was run in triplicate.

The CT value obtained for the target gene in all samples was first normalised with the CT value obtained for the housekeeping gene. The resulting change in CT (Δ*C*_t_) calculated for test samples was then normalised with the Δ*C*_t_ calculated for the control sample, giving a ΔΔ*C*_t_ value. The gene expression ratio was calculated using 2^−ΔΔ*C*t^, providing a fold increase or decrease in gene expression compared to the control sample. Gene fold changes ≥±2 were considered significant.

### 4.7. Western Blot Analysis

Protein was harvested from cells in 100 µL lysis buffer (1% (*v*/*v*) SDS, 30 mM Na_3_VO_4_, 1 µM DTT, protease inhibitor cocktail (Sigma-Aldrich) and phenylmethanosulfonylfluoride (PMSF)) prepared in 1xPBS as previously described [52].

Equivalent amounts of protein (30–80 μg) were mixed with 5 µL loading buffer (NuPAGE^®^ LDS Sample Buffer (4X)) and the volume adjusted to 20 µL with lysis buffer. Samples were mixed for 15 min at 20 rpm followed by brief centrifugation and separated by 4–12% sodium dodecyl sulphate polyacrylamide gel electrophoresis (SDS-PAGE) (NuPAGE^®^ Novex^®^ Bis-Tris Mini Gels; Thermo Fisher) followed by electrophoretic transfer to a nitrocellulose membrane using an I-Blot transfer system (Invitrogen) following manufacturer’s instructions. Transfer efficiency was determined by staining the blots with Ponceau S (0.1% (*w*/*v*) in 5% acetic acid) (Sigma-Aldrich) for 15 s, prior to rinsing in distilled water and probing for relevant protein expression using appropriate primary antibodies as previously described [52]. Membranes were probed with primary antibodies to detect Cx43 (Rivedal polylclonal antibody 1:2000 dilution, kindly gifted by Edward Leithe [53]), GAPDH (mouse monoclonal antibody, Santa Cruz (LOCATION) (1:5000 dilution)) and pSmad3 (rabbit polyclonal antibody Abcam (Cambridge, UK) (1:2000 dilution)) expression as appropriate. Secondary antibodies were IRDye^®^ 800CW goat anti-rabbit IgG or IRDye^®^ 680CW goat anti-mouse IgG (Licor 1:15,000 dilution) as appropriate. Blots were developed by exposing the image for a period of 15 s to 5 min according to the intensity of the signal using an Odyssey FC Dual Mode Licor imaging system (LI-COR Biosceinces UK Ltd, Lincoln, UK). Densitometric values were quantified using the Odyssey software. To enable normalisation of the blots and comparison of the effect of different treatments on protein expression, the intensity of the protein bands were compared to the house keeping protein.

### 4.8. Statistical Analysis

Experiments were performed in triplicate per setting and on three separate occasions with at least 2 different patient samples. Results were compiled in GraphPad Prism software (La Jolla, San Diego, CA, USA) and all data is expressed as mean ± SEM unless otherwise stated. Statistical tests were performed on the data using Student’s unpaired *t*-test or one-way ANOVA and Dunnett’s post-test as appropriate, with statistical significance inferred at *p* < 0.05.

## 5. Conclusions

In conclusion, we provide an in-depth study on the comparative effects of Gap27 with a Cx43-SiRNA knockdown approach to improve wound healing and identify significant differences in the cell signalling pathways that are controlled by Cx43 in fibroblasts and keratinocytes. Further work is now warranted to define the molecular pathways by which Cx43 exerts its effects in the skin which will aide in identifying new therapeutic strategies and applications for specific types of wounds.

## Figures and Tables

**Table 1:** Summary of the effect of 100 nM Gap27 (Gap27) and Cx43-SiRNA (SiRNA) on cellular events related to wound closure in JFF, AK, ADF and HDNF cells. Hemichannel activity was monitored following 90 min exposure to peptide and 15 min challenge with Calcium free media. All other assays were recorded 24 h post scrape wounding in the presence or absence of 100 nM Gap27 or Cx43-SiRNA. *n* = 3 in all cases. ND: not determined; NE: no effect; ↑ enhanced effect; ↓ inhibitory effect. For details of experimental design and statistics see text and figures.

Cell Type	Treatment	HC	Migration	Cx43 Protein	Proliferation	TGF-β1	pSmad3
JFF	SiRNA	ND	↑	↓	NE	↑	↑
	Gap27	↓	↑	↓	NE	↑	↑
AK	SiRNA	ND	↑	↓	NE	NE	NE
	Gap27	↓	↑	↓	NE	NE	NE
ADF	SiRNA	ND	↑	↓	↑	↑	↑
	Gap27	↓	NE	NE	NE	NE	↑
HDNF	SiRNA	ND	↑	↓	↑	NE	NE
	Gap27	↓	NE	NE	↑	NE	NE

## Supplementary Materials

Supplementary materials can be found at http://www.mdpi.com/1422-0067/19/2/604/s1.

## Abbreviations

Cx	connexin
CMP	connexin mimetic peptide
CMC	connexin mediated communication
JFF	human juvenile foreskin derived fibroblasts
HNDF	human neonatal dermal fibroblasts
AF	adult dermal derived fibroblasts
AK	adult keratinocytes
SFM	serum free media
