# Supplementary material for: The Connexin Mimetic Peptide Gap27 and Cx43-Knockdown Reveal Differential Roles for Connexin43 in Wound Closure Events in Skin Model Systems
Source: Int J Mol Sci. 2018 Feb 18;19(2):604. doi: 10.3390/ijms19020604 (PMC5855826; doi:10.3390/ijms19020604)
Supplement: Supplementary file 1 [file ijms-19-00604-s001.pdf]

Supplementary

The Connexin Mimetic Peptide Gap27 and Cx43-Knockdown Reveal Differential Roles for Connexin43 in Wound Closure Events in Skin Model Systems

Chrysovalantou Faniku [1], Erin O'Shaughnessy [1], Claire Lorraine [1], Scott R. Johnstone [1,2,3], Annette Graham [1], Sebastian Greenhough [1,4] and Patricia E.M. Martin [1,*]

[1] Department of Life Sciences, School of Health and Life Sciences, Glasgow Caledonian University,
Glasgow G4 0BA, UK; Chrysovalantou.Faniku@gcu.ac.uk (C.F.); Erin.OShaughnessy@gcu.ac.uk (E.O.); clairelorraine@hotmail.co.uk (C.L.); srj6n@eservices.virginia.edu (S.R.J.); ann.graham@gcu.ac.uk (A.G.); S.Greenhough@beatson.gla.ac.uk (S.G.)

[2] Robert M. Berne Cardiovascular Research Center, University of Virginia School of Medicine, P.O. Box 801394, Charlottesville, VA 22908, USA

[3] Institute of Cardiovascular and Medical Sciences, College of Medical, Veterinary and Life Sciences, University of Glasgow, Glasgow G12 8TT, UK

[4] Current Address: Cancer Research UK Beatson Institute, Garscube Estate, Switchback Road, Bearsden, Glasgow G61 1BD, UK

* Correspondence: patricia.martin@gcu.ac.uk; Tel.: +44-141-331-3726

Table S1. qRT-PCR primers.

Gene ID	Forward	Reverse	Probe
Cx43	5′ACTGGCGACAGAA ACAATTCTTC 3′	5′TTCTGCACTGTAATTA GCCCA GTT 3′	5′56-FAMCGCAATTACAACAAGCAAG CAAGTGAGC 36-TAMsp 3′
GAPDH	5′CACATGGCCTCCAA GGAGTAA 3′	5′TGAGGGTCTCTCTCTTC CTCTTGT 3′	5′56-FAMCTGGACCACCAGCCCCAGC AAG 36-TAMsp 3′
Ki67	5′ TGTCGTCGT TTG TTTGCCTAT 3′	5′CTCATCCATTCATTCGT GTTTACC 3′	5′56-FAMAGTTGTTCCTGCCACCGTGC CCTG 36-TAMsp 3′
TGFb1	5′CACTCCCACTCCCT CTCTC 3′	5′GTCCCCTGTGCCTTGA TG 3′	5′56-FAMTCTCTGCCTCCTCCTGCCTGT CTGC 36-TAMsp 3′
